# Supplementary material for: Tox21mer, A transformer foundation model for Tox21 high-throughput concentration–response curves data
Source: bioRxiv. 2026 Jun 19:2026.06.15.732308. Preprint. [Version 1] doi: 10.64898/2026.06.15.732308 (PMC13308148; doi:10.64898/2026.06.15.732308)
Supplement: Supplement 1 [file media-1.pdf]

Supplementary materials for

Tox21mer, A transformer foundation model for Tox21 high-throughput concentration–response curves data

Table S1. Tox21mer metadata.

Protocol Name	Assay Target	Target Category	Cell Line	Cell Type	Mode	Technology
tox21-ache-p3	AChE (colorimetric)	Neurotoxicity	SH-SY5Y	Neuroblast	Inhibition	Colorimetric
tox21-ache-p4	AChE (fluorescent)	Neurotoxicity	SH-SY5Y	Neuroblast	Inhibition	Fluorescence
tox21-ache-p5	AChE	Neurotoxicity	NA	Biochemical	Inhibition	Colorimetric
tox21-adrb2-agonist-p1	ADRB2 agonist	GPCR	CHO	Hamster	Agonist	Fluorescence
tox21-adrb2-antagonist-p1	ADRB2 antagonist	GPCR	CHO	Hamster	Antagonist	Fluorescence
tox21-ahr-p1	AhR	NR	HepG2	Liver	Agonist	Luciferase
tox21-ap1-agonist-p1	AP-1 agonist	SR	ME-180	Cervical Cancer	Agonist	Beta-lactamase
tox21-ar-bla-agonist-p1	AR-BLA agonist	NR	HEK293	Kidney	Agonist	Beta-lactamase
tox21-ar-bla-antagonist-p1	AR-BLA antagonist	NR	HEK293	Kidney	Antagonist	Beta-lactamase
tox21-are-bla-p1	ARE	SR	HepG2	Liver	Agonist	Beta-lactamase
tox21-ar-mda-kb2-luc-agonist-p1	AR-MDA agonist	NR	MDA-MB-453	Breast Cancer	Agonist	Luciferase
tox21-ar-mda-kb2-luc-agonist-p3	AR-MDA agonist (with antagonist)	NR	MDA-MB-453	Breast Cancer	Agonist	Luciferase
tox21-ar-mda-kb2-luc-antagonist-p1	AR-MDA antagonist	NR	MDA-MB-453	Breast Cancer	Antagonist	Luciferase
tox21-ar-mda-kb2-luc-antagonist-p2	AR-MDA antagonist (lower agonist)	NR	MDA-MB-453	Breast Cancer	Antagonist	Luciferase
tox21-aromatase-p1	Aromatase	SR	MCF-7	Breast Cancer	Inhibition	Luciferase
tox21-car-agonist-p1	CAR agonist	NR	HepG2	Liver	Agonist	Luminescence
tox21-car-antagonist-p1	CAR antagonist	NR	HepG2	Liver	Antagonist	Luminescence
tox21-casp3-cho-p1	Caspase-3/7	Cytotoxicity	CHO	Hamster	Activation	Luminescence
tox21-casp3-hepg2-p1	Caspase-3/7	Cytotoxicity	HepG2	Liver	Activation	Luminescence
tox21-chrm1-p1	CHRM1 agonist and antagonist	GPCR	CHO	Hamster	Agonist and Antagonist	Fluorescence
tox21-cre-agonist-p1	CRE agonist	SR	HEK293	Kidney	Agonist	Beta-lactamase
tox21-cre-antagonist-p1	CRE antagonist	SR	HEK293	Kidney	Antagonist	Beta-lactamase
tox21-drd2-agonist-p1	DRD2 agonist	GPCR	HEK293	Kidney	Agonist	Fluorescence
tox21-drd2-antagonist-p1	DRD2 antagonist	GPCR	HEK293	Kidney	Antagonist	Fluorescence
tox21-dt40-p1-100	Cell viability	Gene Tox	DT40	Chicken	Cytotoxicity	Luminescence
tox21-dt40-p1-653	Cell viability	Gene Tox	DT40	Chicken	Cytotoxicity	Luminescence
tox21-dt40-p1-657	Cell viability	Gene Tox	DT40	Chicken	Cytotoxicity	Luminescence
tox21-elg1-luc-agonist-p1	ATAD5	Gene Tox	HEK293	Kidney	Agonist	Luciferase
tox21-erb-bla-antagonist-p1	ER-beta antagonist	NR	HEK293	Kidney	Antagonist	Beta-lactamase
tox21-erb-bla-p1	ER-beta agonist	NR	HEK293	Kidney	Agonist	Beta-lactamase
tox21-er-bla-agonist-p2	ER-BLA agonist	NR	HEK293	Kidney	Agonist	Beta-lactamase
tox21-er-bla-antagonist-p1	ER-BLA antagonist	NR	HEK293	Kidney	Antagonist	Beta-lactamase
tox21-er-luc-bg1-4e2-agonist-p2	ER-BG1 agonist	NR	BG1	Ovarian	Agonist	Luciferase

tox21-er-luc-bg1-4e2-agonist-p4	ER-BG1 agonist (with antagonist)	NR	BG1	Ovarian	Agonist	Luciferase
tox21-er-luc-bg1-4e2-antagonist-p1	ER-BG1 antagonist	NR	BG1	Ovarian	Antagonist	Luciferase
tox21-er-luc-bg1-4e2-antagonist-p2	ER-BG1 antagonist (lower agonist)	NR	BG1	Ovarian	Antagonist	Luciferase
tox21-err-p1	ERR	NR	HEK293	Kidney	Agonist	Fluorescence
tox21-esre-bla-p1	ER stress	SR	HeLa	Cervical Cancer	Agonist	Beta-lactamase
tox21-fxr-bla-agonist-p2	FXR-BLA agonist	NR	HEK293	Kidney	Agonist	Beta-lactamase
tox21-fxr-bla-antagonist-p1	FXR-BLA antagonist	NR	HEK293	Kidney	Antagonist	Beta-lactamase
tox21-gh3-tre-agonist-p1	TR-beta agonist	NR	GH3	Rat pituitary	Agonist	Luciferase
tox21-gh3-tre-antagonist-p1	TR-beta antagonist	NR	GH3	Rat pituitary	Antagonist	Luciferase
tox21-gnrhr-hek293-p2	GnRHR agonist	GPCR	HEK293	Kidney	Agonist	Fluorescence
tox21-gr-hela-bla-agonist-p1	GR-BLA agonist	NR	HeLa	Cervical Cancer	Agonist	Beta-lactamase
tox21-gr-hela-bla-antagonist-p1	GR-BLA antagonist	NR	HeLa	Cervical Cancer	Antagonist	Beta-lactamase
tox21-h2ax-cho-p2	H2AX	Gene Tox	CHO	Hamster	Activation	Fluorescence
tox21-hdac-p1	HDAC	Gene Tox	HCT-116	Colon Cancer	Inhibition	Luminescence
tox21-herg-u2os-p1	hERG	Cardiotoxicity	U2OS	Osteosarcoma	Inhibition	Fluorescence
tox21-hre-bla-agonist-p1	HRE-BLA agonist	SR	ME-180	Cervical Cancer	Agonist	Beta-lactamase
tox21-hse-bla-p1	HSE-BLA	SR	HeLa	Cervical Cancer	Agonist	Beta-lactamase
tox21-htr2a-p1	HTR2A agonist and antagonist	GPCR	CHO	Hamster	Agonist and Antagonist	Fluorescence
tox21-kiss1r-hek293-p2	KISS1R agonist	GPCR	HEK293	Kidney	Agonist	Fluorescence
tox21-kiss1r-wt-p2	KISS1R wild type	GPCR	HEK293	Kidney	Agonist	Fluorescence
tox21-ks-are-p1	Nrf2/ARE	SR	HaCaT	Skin	Agonist	Luciferase
tox21-mitotox-p1	Mitochondria toxicity	SR	HepG2	Liver	Inhibition	Fluorescence
tox21-ms-ache-p2	AChE (with human microsomes)	Neurotoxicity	NA	Biochemical	Inhibition	Colorimetric
tox21-ms-p53-p1	P53 (with rat microsomes)	Gene Tox	HCT-116	Colon Cancer	Agonist	Beta-lactamase
tox21-ms-p53-p2	P53 (with human microsomes)	Gene Tox	HCT-116	Colon Cancer	Agonist	Beta-lactamase
tox21-nfkb-bla-agonist-p1	NFkB agonist	SR	ME-180	Cervical Cancer	Agonist	Beta-lactamase
tox21-p450-1a2-p1	CYP1A2	Metabolism	NA	Biochemical	Inhibition	Luminescence
tox21-p450-2c19-p1	CYP2C19	Metabolism	NA	Biochemical	Inhibition	Luminescence
tox21-p450-2c9-p1	CYP2C9	Metabolism	NA	Biochemical	Inhibition	Luminescence
tox21-p450-2d6-p1	CYP2D6	Metabolism	NA	Biochemical	Inhibition	Luminescence
tox21-p450-3a4-p1	CYP3A4	Metabolism	NA	Biochemical	Inhibition	Luminescence
tox21-p53-bla-p1	P53	Gene Tox	HCT-116	Colon Cancer	Agonist	Beta-lactamase
tox21-p53-bla-p7	P53	Gene Tox	HCT-116	Colon Cancer	Agonist	Beta-lactamase
tox21-pgc-err-p1	PGC-ERR	NR	HEK293	Kidney	Agonist	Luminescence

tox21-ppard-bla-agonist-p1	PPAR-delta-BLA agonist	NR	HEK293	Kidney	Agonist	Beta-lactamase
tox21-ppard-bla-antagonist-p1	PPAR-delta-BLA antagonist	NR	HEK293	Kidney	Antagonist	Beta-lactamase
tox21-pparg-bla-agonist-p1	PPAR-gamma agonist	NR	HEK293	Kidney	Agonist	Beta-lactamase
tox21-pparg-bla-antagonist-p1	PPAR-gamma antagonist	NR	HEK293	Kidney	Antagonist	Beta-lactamase
tox21-pr-bla-agonist-p1	PR-BLA agonist	NR	HEK293	Kidney	Agonist	Beta-lactamase
tox21-pr-bla-antagonist-p1	PR-BLA antagonist	NR	HEK293	Kidney	Antagonist	Beta-lactamase
tox21-pxr-p1	PXR agonist	NR	HepG2	Liver	Agonist	Luciferase
tox21-rar-agonist-p1	RAR agonist	NR	C3H10T1/2	Murine embryo fibroblast	Agonist	Luciferase
tox21-rar-antagonist-p2	RAR antagonist	NR	C3H10T1/2	Murine embryo fibroblast	Antagonist	Luciferase
tox21-rar-viability-p2	RAR viability	Cytotoxicity	C3H10T1/2	Murine embryo fibroblast	Cytotoxicity	Luciferase
tox21-ror-cho-antagonist-p1	ROR antagonist	NR	CHO	Hamster	Antagonist	Luciferase
tox21-ror-cho-viability-p1	ROR viability	Cytotoxicity	CHO	Hamster	Cytotoxicity	Fluorescence
tox21-rt-viability-hek293-p1-flor_8h_n	Cell viability	Cytotoxicity	HEK293	Kidney	Cytotoxicity	Fluorescence
tox21-rt-viability-hek293-p1-flor_16h_n	Cell viability	Cytotoxicity	HEK293	Kidney	Cytotoxicity	Fluorescence
tox21-rt-viability-hek293-p1-flor_24h_n	Cell viability	Cytotoxicity	HEK293	Kidney	Cytotoxicity	Fluorescence
tox21-rt-viability-hek293-p1-glo_8h_n	Cell viability	Cytotoxicity	HEK293	Kidney	Cytotoxicity	Luminescence
tox21-rt-viability-hek293-p1-glo_16h_n	Cell viability	Cytotoxicity	HEK293	Kidney	Cytotoxicity	Luminescence
tox21-rt-viability-hek293-p1-glo_24h_n	Cell viability	Cytotoxicity	HEK293	Kidney	Cytotoxicity	Luminescence
tox21-rt-viability-hepg2-p1-flor_8h_n	Cell viability	Cytotoxicity	HepG2	Liver	Cytotoxicity	Fluorescence
tox21-rt-viability-hepg2-p1-flor_16h_n	Cell viability	Cytotoxicity	HepG2	Liver	Cytotoxicity	Fluorescence
tox21-rt-viability-hepg2-p1-flor_24h_n	Cell viability	Cytotoxicity	HepG2	Liver	Cytotoxicity	Fluorescence
tox21-rt-viability-hepg2-p1-glo_8h_n	Cell viability	Cytotoxicity	HepG2	Liver	Cytotoxicity	Luminescence
tox21-rt-viability-hepg2-p1-glo_16h_n	Cell viability	Cytotoxicity	HepG2	Liver	Cytotoxicity	Luminescence
tox21-rt-viability-hepg2-p1-glo_24h_n	Cell viability	Cytotoxicity	HepG2	Liver	Cytotoxicity	Luminescence
tox21-rxr-bla-agonist-p1	RXR-BLA	NR	HEK293	Kidney	Agonist	Beta-lactamase

tox21-sbe-bla-agonist-p1	SBE-BLA (TGF-beta) agonist	Developmental Toxicity	HEK293	Kidney	Agonist	Beta-lactamase
tox21-sbe-bla-antagonist-p1	SBE-BLA (TGF-beta) antagonist	Developmental Toxicity	HEK293	Kidney	Antagonist	Beta-lactamase
tox21-shh-3t3-gli3-agonist-p1	Hedgehog agonist	Developmental Toxicity	NIH/3T3	Murine embryo fibroblast	Agonist	Luciferase
tox21-shh-3t3-gli3-antagonist-p1	Hedgehog antagonist	Developmental Toxicity	NIH/3T3	Murine embryo fibroblast	Antagonist	Luciferase
tox21-spec-hek293-p1	Auto Fluorescence	Counter Screen	HEK293	Kidney	Counter	Fluorescence
tox21-spec-hepg2-p1	Auto Fluorescence	Counter Screen	HepG2	Liver	Counter	Fluorescence
tox21-trhr-hek293-p1	TRHR agonist and antagonist	GPCR	HEK293	Kidney	Agonist and Antagonist	Fluorescence
tox21-tshr-agonist-p1	TSHR agonist	GPCR	HEK293	Kidney	Agonist	Fluorescence
tox21-tshr-antagonist-p1	TSHR antagonist	GPCR	HEK293	Kidney	Antagonist	Fluorescence
tox21-tshr-wt-p1	TSHR wild type	GPCR	HEK293	Kidney	Agonist	Fluorescence
tox21-vdr-bla-agonist-p1	VDR-BLA agonist	NR	HEK293	Kidney	Agonist	Beta-lactamase
tox21-vdr-bla-antagonist-p1	VDR-BLA antagonist	NR	HEK293	Kidney	Antagonist	Beta-lactamase

Tox21 Data Curation and Filtering

We began with 7,947,446 Tox21 concentration–response curves and applied a series of structural, data-quality, and assay-content filters. First, we removed curves associated with very small chemical structures (10,506 with fewer than three heavy atoms), ambiguous concentration annotations [3,073 lacking a `curve_class2(cc2)` label or with `cc2` > 4], and low-purity samples (2,026,038 with purity other than A or B). We then filtered on assay content encoded in SAMPLE_DATA_TYPE, excluding raw channel readouts (2,770,160), viability readouts (1,748,332), and FITC- or rhodamine-labeled channels (35,328 each). These endpoints were excluded because they are either handled differently in downstream analyses—for example, ratio metrics derived from paired channels such as ch1 and ch2, or used as counterscreens, such as viability measurements in mechanistic agonist/antagonist classification. After all filtering steps, 2,499,594 curves remained for the downstream analysis.

Data Quality Control
We applied the following quality-control filters to ensure data reliability:

1. Valid-response threshold: each curve was required to have at least twelve valid (non-missing) response measurements out of 15 concentration points.

2. Consistency enforcement: for curves classified as `Curve_class2(cc2)=4` (flat/inactive), we enforced consistency by setting Assay_outcome to "inactive" and AC_{50} to NaN. Rows where `cc2=4` and the recorded Assay_outcome were inconsistent were demoted to inconclusive.

3. Sign-consistency check: agonist-labelled replicates with negative cc2 and antagonist-labelled replicates with positive `cc2` (994 and 1,023 rows respectively) were demoted to inconclusive with AC_{50} set to NaN.

Branch Structure
The 102 assays were partitioned into mechanistic and non-mechanistic branches according to assay mode (Table S1). This branch-level formulation was designed to enable assay-mode-specific prediction consistent with the biological interpretation of the underlying readout. Mechanistic assays were modeled as a three-class classification task with labels agonist, antagonist, and inactive, whereas non-mechanistic assays were treated as a binary classification task with labels active and inactive. Mechanistic-branch outcomes containing the term "cytotoxic" were excluded because potential cytotoxicity could interfere with the primary mechanistic response, and such responses can resemble antagonist-like profiles. Inconclusive agonists were merged into the agonist class and inconclusive antagonists into the antagonist class, whereas records labeled only as inconclusive were excluded.

Outcome-label collapsing

The eight original assay outcome strings ("active agonist", "active antagonist", "inactive", "inconclusive", "inconclusive agonist", "inconclusive agonist (cytotoxic)", "inconclusive antagonist", "inconclusive antagonist (cytotoxic)" recorded in Tox21 records are mapped to five collapsed classes (Table S2).

For the mechanism branch, only the three primary classes - agonist, antagonist, and inactive - are used as supervised outcome labels during both pretraining and probing. The cytotoxic and inconclusive classes are excluded from outcome and AC_{50} supervision, but their rows are retained for the masked-response reconstruction objective. For the non-mechanism branch, only the two primary classes - active and inactive - are used as supervised outcome labels during both pretraining and probing.

Table S2. Outcome-label collapsing strategy

Original Tox21 outcome string	Collapsed class	Supervised?	Notes
`active agonist`<br>`inconclusive agonist`	Agonist	Yes	Both rows treated as the active-agonist class for outcome supervision.
`active antagonist`<br>`inconclusive antagonist`	Antagonist	Yes	Both rows treated as the active-antagonist class for outcome supervision.
`inactive`	Inactive	Yes	Used as the inactive class for outcome supervision.
`inconclusive agonist (cytotoxic)`<br>`inconclusive antagonist (cytotoxic)`	Cytotoxic	No	Excluded from outcome and AC_{50} supervision; cytotoxicity confounds the receptor signal.
`inconclusive`	Inconclusive	No	Excluded from outcome and AC_{50} supervision; retained for masked-response pretraining only.

Target Variables

We prepared three complementary prediction targets: (1) concentration response values: normalized continuous responses at each concentration point for masked-response prediction; (2) assay outcome classification: agonist, antagonist, and inactive for the mechanism branch and active and inactive for the non-mechanism branch; (3) AC_{50} regression, $\log_{10}$-transformed AC_{50} values for compounds with active responses (zero AC_{50} values are treated as NaN).

Model architecture

Categorical embeddings

Six assay descriptors: `Protocol_name`, `Target_category`, `Cell_type`, `Mode`, `Technology`, and `is_ratio`, each use a learned embedding table. In our implementation, `Protocol_name` uses embedding width 64 and the other five use width 32; each table is linearly projected to the model hidden size 768, and the six projected vectors are summed to form a single assay-context vector `assay_ctx`.

Dose–response token encoding

For each of the 15 concentration positions we build a token as follows:

Dose (**concentration**): $\log_{10}$ concentration is passed through a two-layer `MLP` (1 → 256 → 768) with `ReLU` between layers. Response: the per-dose normalized response is passed through a separate two-layer `MLP` (1 → 256 → 768) with `ReLU`.

Token: dose (concentration) and response embeddings are added to `assay_ctx` (broadcast along the dose axis) and passed through LayerNorm:
`token_i=LayerNorm(assay_ctx+dose_emb_i+response_emb_i)`
(An ablation can omit `assay_ctx` from DR tokens; the default includes it.)

Special tokens

A learnable [`CLS`] vector is added to `assay_ctx` and LayerNorm-normalized: [`CLS`] = LayerNorm(`CLS_base` + `assay_ctx`). At masked pretraining positions, the response channel is replaced by a learnable [`MASK`] embedding before the response `MLP` output is combined into the token.

Transformer encoder

The 16-token sequence ([`CLS`] plus 15 dose–response tokens) is encoded by a `TransformerEncoder`. In the released configuration we use `ASSAY_NUM_LAYERS = 6` encoder layers (six stacked `TransformerEncoderLayer` blocks), eight attention heads, hidden size 768, feed-forward size 4 × 768 = 3,072, dropout 0.1, pre-norm (`norm_first=True`), and `GELU` activation inside the encoder blocks.

Masked response prediction: a linear 768 → 1 head applied to each DR (dose response) token. Outcome classification: branch-specific linear heads applied during Phase 1 when auxiliary supervision is enabled ($\lambda = 0.1$). For the mechanism branch, a linear 768 → 3 head predicts inactive, agonist, or antagonist. For the non-mechanism branch, a linear 768 → 2 head predicts inactive or active. Cytotoxic and inconclusive rows are excluded from the supervised outcome loss.

Branch probe heads (evaluation only)

After Phase 1, we attach two linear probe heads on the frozen CLS vector: a 3-class mechanism head (agonist, antagonist, inactive) and a 2-class non-mechanism head (active, inactive). These are for evaluation, not Phase 1 optimization.

Training procedure

Phase 1: backbone pre-training

Each step randomly masks 20% of valid response positions; masked sites use the [MASK] embedding. The model is trained to reconstruct normalized responses at masked positions (masked MSE). Unknown / inconclusive outcomes (label −1) are excluded from supervised terms; supervised terms also restrict to rows whose cc2 lies in the allowed replicate-aggregation whitelist for the chosen `use_cc2` mode (strict vs loose). Under `cc2=strict`, the allowed set is (±1.1, ±1.2, ±2.1, ±2.2, ±4.0); loose adds additional cc2 classes, i.e., ±1.3, ±1.4, ±2.3, ±2.4, ±3.0, which is the full valid cc2 list.

Optimization: AdamW, learning rate 1×10^{-4}, weight decay 1×10^{-4}, 50 epochs. Default batch size is 4,096.

Checkpoint selection

For all pretraining variants, the backbone checkpoint was selected based on the lowest validation masked-response MSE. Validation outcome accuracy was recorded for monitoring purposes but was not used for checkpoint selection. This ensured that model selection remained consistent across variants and was tied to the shared reconstruction objective.

Phase 2: outcome classification probe

We load assay_backbone_best.pt, freeze the backbone, and train only the two probe heads for 5 epochs (AdamW, LR 1×10^{-3}, weight decay 1×10^{-4}). Mechanism branch: focal loss with $\gamma = 2.0$ and class α vector [1, 1, 1] for (agonist, antagonist, inactive). Non-mechanism branch: cross-entropy. Checkpoint selection: maximize validation 0.5·(macro-F1_mechanism + binary-F1_non-mechanism).

Phase 3: AC_{50} regression probe

Using the same backbone checkpoint selected by validation masked-response MSE, we froze the backbone and trained the built-in CLS AC50 head (head_ac50) for 5 epochs using AdamW with a learning rate of 10^{-3}. The training criterion is mean squared error on $\log_{10} AC_{50}$ for rows with finite ac50_log (raw $AC_{50} > 0$ before the log).

Phase 4: AC_{50} probe evaluation

AC_{50} probe predictions were evaluated per-protocol and overall on the validation and test splits using RMSE, MAE, R^2, and Pearson correlation. Sample-weighted aggregates across the 102 protocols were additionally computed.

Phase 5: foundation model

For deployment, we retrained Tox21mer on the full quality-controlled dataset, including all compounds from the original training, validation, and test splits, using the same architecture and hyperparameters as in the split-based pretraining stage. The resulting full-data checkpoint was designated as the final foundation model for downstream applications. Because this retraining step used the entire dataset, no held-out set remained for model selection at this stage; accordingly, unbiased estimates of generalization remain those obtained from the original split-based evaluation. From the final full-data model, we extracted a 768-dimensional [`CLS`] embedding for each individual curve and then averaged embeddings across technical replicates for each unique (chemical, protocol) pair to generate the representations used in downstream analyses.

Implementation details & computing

All models were implemented in PyTorch 2.x. Random seeds were fixed (seed= 42) for all stochastic operations, and deterministic CUDA behavior was enabled by setting `torch.backends.cudnn.deterministic = True` and `torch.backends.cudnn.benchmark = False`. DataLoader workers were seeded using a `worker_init_fn` aligned with `torch.initial_seed()`. For full bitwise reproducibility on GPU, the environment variable `CUBLAS_WORKSPACE_CONFIG=:4096:8` was set. Mixed-precision training was used where applicable, and 8–16 DataLoader workers were employed to improve throughput across multiple GPUs.

All training runs were performed on NVIDIA A100-SXM4 GPUs with either 40 GB or 80 GB of memory. A single Phase 1 training run (50 epochs; 1,736,846 training rows; batch size 4.096) required approximately 6 hours. Phase 5 retraining on the full dataset required an additional approximately 7 hours. The probe stages (Phases 2–4) together required approximately 15 minutes on a single GPU. Each `MLP` baseline completed in less than 2 hours.

Additional Results

Results for `CC2=Loose`

As mentioned before, we defined two cc2 groupings: `cc2 strict` and `cc2 loose`. The `cc2 strict` group comprised the original Tox21 classes (-1.1, -1.2, -2.1, -2.2, 1.1, 1.2, 2.1, 2.2, and 4), whereas the cc2 loose group included 1.1, 1.2, 1.3, 1.4, 2.1, 2.2, 2.3, 2.4, 3.0, 4.0, -1.1,-1.2, -1.3, -1.4, -2.1, -2.2, -2.3, -2.4. In the main manuscript, all probing analyses and model training were conducted using `cc2=strict`. Below, we provide summary results for `cc2=loose`.

We compared branch-classification performance under `cc2=strict` (**Table 3a**, **3b**) versus `cc2= loose` (**Table S3a** and **3b**). Overall, the strict setting performed substantially better: the composite score increased from 0.9607 under `cc2 =loose` to 0.9891 under

`cc2=strict`, a difference of 2.84 points. Most of this gap was driven by the mechanism branch, where macro-F1 declined from 0.9848 to 0.9467 when moving from `strict` to `loose`. By comparison, the non-mechanism branch showed a smaller decrease, with binary F1 falling from 0.9935 to 0.9747. Test-set support also changed, particularly for mechanism-active classes—because the `loose` definition retains additional cc2 patterns excluded under the `strict` definition (e.g., agonist/antagonist support increased from 9,302 / 7,180 under `strict` to 12,158 / 10,305 under `loose`). Thus, part of the difference reflects the broader and more challenging label space under cc2 = `loose`, rather than a change in model performance on an identical set of rows.

Among mechanism classes, agonist prediction was affected most strongly by the `loose` setting. Agonist F1 decreased from 0.9779 to 0.8988, driven primarily by a drop in precision from 0.9810 to 0.8251, while recall remained high (0.9750 to 0.9868). This pattern is consistent with increased overcalling of inactive compounds as agonists. For example, the confusion matrix shows 144,674 correct inactive predictions under `loose` versus 147,426 under strict, with 2,506 + 566 inactive rows misclassified as agonist or antagonist under `loose` compared with only 162 + 158 under strict. Antagonist performance also worsened under the `loose` setting, with F1 declining from 0.9787 to 0.9535.

The non-mechanism branch showed the same general trend, although at a smaller scale. Accuracy declined from 0.9899 under `strict` to 0.9643 under loose, accompanied by more active/inactive confusion: 778 active→inactive and 2,503 inactive→active errors under loose, compared with 218 and 638, respectively, under `strict`. Although the dominant error type differed, the total error burden was clearly larger under the `loose` condition.

Ranking metrics were consistent with these classification results. Per-class PR-AUC and ROC-AUC remained high in both settings but were uniformly better under `cc2=strict` (e.g., non-mechanism active PR-AUC: 0.9981 under strict vs. 0.9913 under `loose`). These results suggest that expanding the allowable cc2 set introduces cases that are less cleanly separable in feature space, rather than merely affecting metric reporting. Overall, `cc2 =loose` provides broader curve-type coverage but at the cost of noticeably reduced branch-classification performance, with the mechanism branch and especially agonist versus inactive discrimination showing the greatest degradation.

Table S3a. Test data mechanism and non-mechanism branch classification reports for `cc2=loose`.

	Mechanism branch			
	Precision	Recall	F1-score	Support
Agonist	0.8251	0.9868	0.8988	12,158
Antagonist	0.9445	0.9627	0.9535	10,305
Inactive	0.9966	0.9792	0.9878	147,746

Accuracy			0.9958	170,209
Macro Avg	0.9221	0.9763	0.9848	170,209
Weighted Avg	0.9812	0.9788	0.9958	170,209
		**Non-mechanism branch**		
Active	0.9102	0.9703	0.9393	26,163
Inactive	0.9878	0.9619	0.9747	65,651
Accuracy			0.9899	91,814
Macro Avg	0.9490	0.9661	0.9570	91,814
Weighted Avg	0.9657	0.9643	0.9646	91,814

Table S3b. Per-class PR-AUC and ROC-AUC on the held-out test set for `cc2 =loose`.

	Branch Class	PR-AUC	ROC-AUC	Support
mechanism	Agonist	0.9899	0.9989	12,158
	Antagonist	0.9922	0.9993	10,305
	Inactive	0.9997	0.9981	147,746
Non-mechanism	Active	0.9913	0.9959	26,163

We also retrained AC_{50} supervision for all curves with `cc2=loose` using an inverse weighted MSE loss as for the `cc2=strict` curves.

In the weighted AC_{50} experiment, the CC2-restricted setting (`cc2=strict`) outperformed `cc2=loose` across both global (**Table S4**) and tail-focused metrics once CC2 gating was properly enforced during AC_{50} training and evaluation (**Figure S2**). Under the strict setting, test performance was consistently stronger across all core regression metrics: R^2 = 0.8738 versus 0.7659, Pearson = 0.9423 versus 0.8981, MAE = 0.2011 versus 0.2912, and RMSE = 0.2894 versus 0.4088. Validation performance also improved substantially, with the best validation MSE decreasing from 0.1632 under loose to 0.0800 under strict.

Importantly, this comparison reflects different row pools by design (n = 52,417 for `cc2=strict` versus 83,143 for `cc2=loose`), because the strict setting excludes Curve_class2 patterns that are retained under the loose definition. Despite using the smaller and more constrained dataset, the strict model showed better calibration in the low-AC_{50} regime, with lower low-tail MAE (0.733 versus 1.034) and a smaller low-tail residual mean (0.733 versus 1.004), indicating reduced positive bias for highly potent compounds. Overall, these weighted results support the conclusion that, for AC_{50} regression, the stricter `cc2` definition provides a cleaner supervision subset and yields materially better predictive performance than the broader loose setting.

Table S4. Comparison in AC_{50} supervision.

Category	N	R^2	Pearson	mae	rmse	Low tail mae
`cc2=strict`	52,417	0.87	0.94	0.201	0.289	0.257
`cc2=loose`	83,143	0.77	0.90	0.291	0.409	1.034

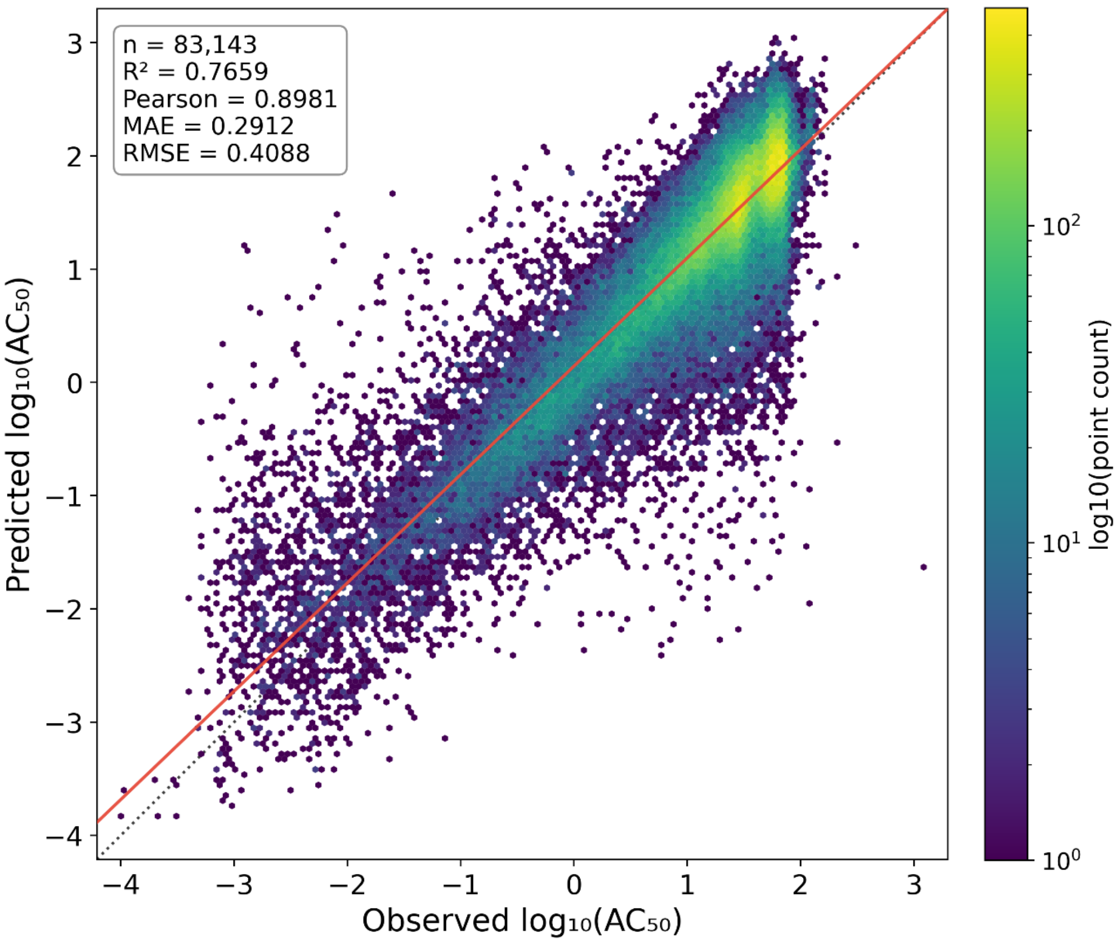

Figure S2. Predicted $\log_{10}(AC_{50})$ vs observed $\log_{10}(AC_{50})$ values for all test curves with valid AC_{50} values and `cc2=loose` (Pearson and Spearman ρ = ~0.89).

As expected, the separation among the different `cc2` classes was less well defined than in the `cc2=loose` setting (**Figure S2**).

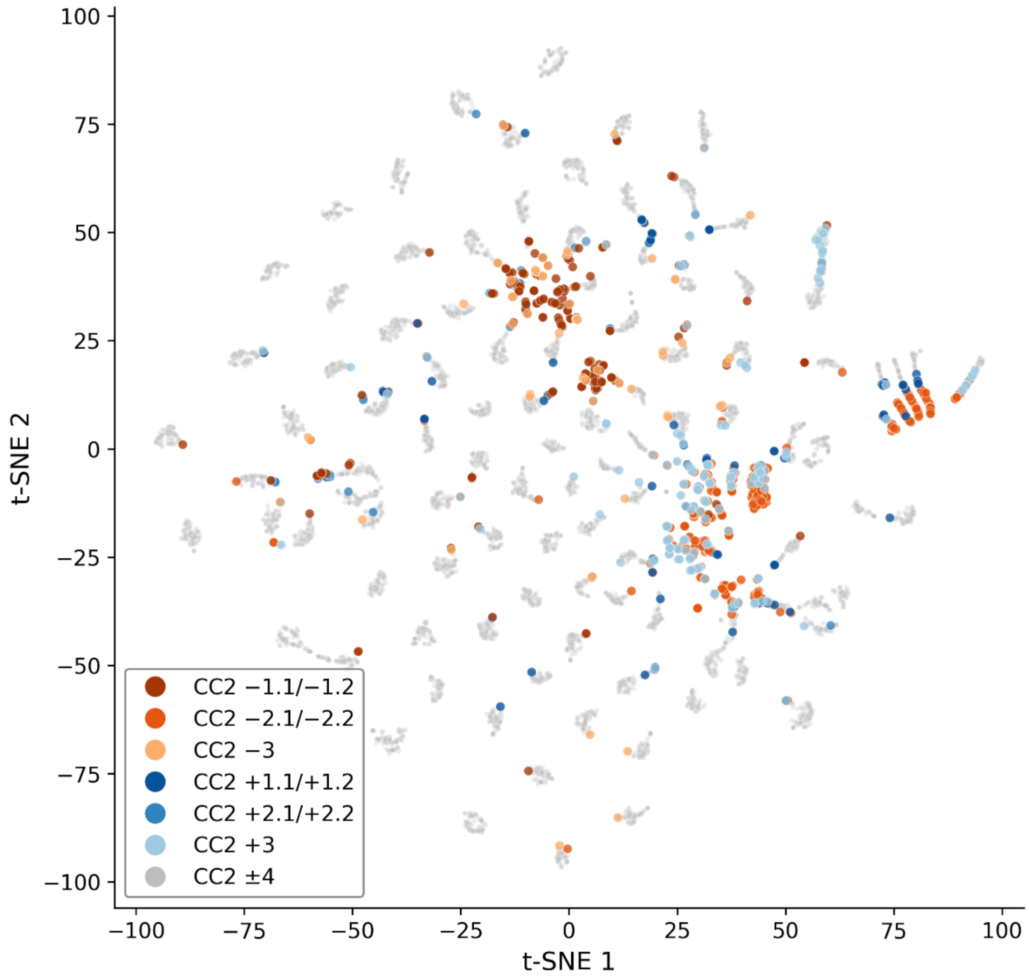

Figure S3. Two-dimensional t-SNE projection of the 768-dimensional embeddings for test curves shown for a stratified subsample of 8,000 of the 394,983 unique (SMILES, protocol) embeddings. The full set of curve class 2 (cc2) labels represented in the model was (1.1, 1.2, 1.3, 1.4, 2.1, 2.2, 2.3, 2.4, 3.0, -1.1, -1.2, -1.3, -1.4, -2.1, -2.2, -2.3, -2.4, -3.0, 4.0). For display purposes, these labels were consolidated into seven groups, shown in the legend.

Table S4. AC_{50} Performance Metrics on Test Curves Using Inverse-Weighted MSE Loss During Training.

Protocol name	n_samples	rmse	mae	R^2	Pearson ρ
tox21-pr-bla-agonist-p1	143	0.2060	0.1611	0.9763	0.9898
tox21-er-luc-bg1-4e2-agonist-p4	177	0.2224	0.1676	0.9720	0.9867
tox21-ar-mda-kb2-luc-agonist-p3	252	0.2377	0.1873	0.9693	0.9847
tox21-er-bla-agonist-p2	297	0.2383	0.1823	0.9655	0.9838
tox21-gr-hela-bla-agonist-p1	315	0.2671	0.1825	0.9551	0.9775
tox21-dt40-p1-657	1289	0.2043	0.1586	0.9511	0.9861
tox21-erb-bla-p1	152	0.3057	0.2285	0.9497	0.9793
tox21-dt40-p1-653	1309	0.2069	0.1585	0.9472	0.9845

tox21-ar-mda-kb2-luc-agonist-p1	190	0.3440	0.2701	0.9404	0.9776
tox21-p450-2c19-p1	1756	0.1692	0.1121	0.9358	0.9758
tox21-drd2-antagonist-p1	171	0.2973	0.1919	0.9351	0.9704
tox21-fxr-bla-antagonist-p1	480	0.2231	0.1438	0.9328	0.9672
tox21-dt40-p1-100	1293	0.2303	0.2008	0.9316	0.9901
tox21-pr-bla-antagonist-p1	1229	0.1692	0.1354	0.9305	0.9798
tox21-aromatase-p1	893	0.2773	0.2076	0.9299	0.9738
tox21-p450-3a4-p1	1451	0.1815	0.1391	0.9269	0.9709
tox21-er-luc-bg1-4e2-agonist-p2	806	0.2716	0.1870	0.9252	0.9660
tox21-ar-mda-kb2-luc-antagonist-p1	780	0.3126	0.2186	0.9179	0.9641
tox21-ror-cho-antagonist-p1	828	0.2179	0.1574	0.9173	0.9647
tox21-rxr-bla-agonist-p1	521	0.2316	0.1706	0.9145	0.9579
tox21-rar-agonist-p1	386	0.2878	0.2178	0.9124	0.9567
tox21-casp3-cho-p1	130	0.2393	0.1879	0.9114	0.9623
tox21-p450-2d6-p1	1435	0.2129	0.1735	0.9058	0.9822
tox21-shh-3t3-gli3-antagonist-p1	1139	0.2435	0.1772	0.9033	0.9659
tox21-casp3-hepg2-p1	327	0.2278	0.1638	0.9010	0.9538
tox21-ar-bla-agonist-p1	309	0.4131	0.3188	0.8986	0.9613
tox21-sbe-bla-antagonist-p1	474	0.2597	0.2174	0.8952	0.9820
tox21-p450-1a2-p1	2174	0.1634	0.1358	0.8944	0.9788
tox21-car-antagonist-p1	1297	0.2241	0.1674	0.8910	0.9533
tox21-tshr-wt-p1	38	0.5135	0.4238	0.8864	0.9729
tox21-ks-are-p1	574	0.2861	0.2073	0.8818	0.9502
tox21-ar-mda-kb2-luc-antagonist-p2	1211	0.2903	0.1772	0.8798	0.9551
tox21-ache-p4	94	0.2290	0.1751	0.8785	0.9549
tox21-rt-viability-hek293-p1-glo_16h_n	252	0.2142	0.1431	0.8757	0.9450
tox21-pparg-bla-antagonist-p1	501	0.2570	0.1902	0.8746	0.9381
tox21-mitotox-p1	1115	0.2385	0.1488	0.8642	0.9336
tox21-ache-p5	62	0.2384	0.1845	0.8641	0.9554
tox21-er-luc-bg1-4e2-antagonist-p2	827	0.2805	0.1978	0.8612	0.9477
tox21-err-p1	2406	0.2505	0.1632	0.8585	0.9332
tox21-er-luc-bg1-4e2-antagonist-p1	631	0.3415	0.2446	0.8573	0.9434
tox21-rar-antagonist-p2	1021	0.2845	0.2148	0.8552	0.9482
tox21-gh3-tre-antagonist-p1	1008	0.2765	0.2285	0.8506	0.9696
tox21-cre-agonist-p1	39	0.4506	0.3338	0.8499	0.9346
tox21-gr-hela-bla-antagonist-p1	635	0.3784	0.2633	0.8487	0.9221
tox21-h2ax-cho-p2	327	0.2816	0.1766	0.8463	0.9278
tox21-ppard-bla-antagonist-p1	291	0.3627	0.2336	0.8389	0.9366
tox21-erb-bla-antagonist-p1	865	0.2738	0.1455	0.8377	0.9171
tox21-chrm1-p1	293	0.4185	0.2928	0.8353	0.9216
tox21-tshr-agonist-p1	273	0.2904	0.2124	0.8346	0.9256
tox21-herg-u2os-p1	576	0.2328	0.1908	0.8285	0.9693
tox21-ahr-p1	588	0.2566	0.1877	0.8248	0.9248
tox21-pxr-p1	1347	0.2445	0.1638	0.8244	0.9190
tox21-esre-bla-p1	228	0.4998	0.3604	0.8227	0.9084
tox21-rt-viability-hek293-p1-glo_8h_n	181	0.1675	0.1246	0.8205	0.9499
tox21-htr2a-p1	654	0.4513	0.3455	0.8199	0.9491
tox21-pparg-bla-agonist-p1	410	0.3413	0.2544	0.8036	0.9006

tox21-p450-2c9-p1	1892	0.2242	0.1730	0.8014	0.9421
tox21-ap1-agonist-p1	427	0.2771	0.2224	0.7961	0.9416
tox21-are-bla-p1	922	0.2874	0.2256	0.7956	0.9286
tox21-rt-viability-hek293-p1-glo_24h_n	287	0.2955	0.1696	0.7932	0.9229
tox21-car-agonist-p1	859	0.2641	0.2137	0.7810	0.9375
tox21-p53-bla-p7	132	0.3727	0.2654	0.7799	0.9216
tox21-cre-antagonist-p1	436	0.2625	0.1896	0.7777	0.9217
tox21-ar-bla-antagonist-p1	905	0.3579	0.2486	0.7728	0.9328
tox21-ache-p3	335	0.2996	0.2317	0.7713	0.9485
tox21-ror-cho-viability-p1	438	0.3325	0.2177	0.7708	0.9085
tox21-gnrhr-hek293-p2	195	0.5156	0.3874	0.7707	0.8791
tox21-p53-bla-p1	461	0.4105	0.2574	0.7705	0.9002
tox21-vdr-bla-antagonist-p1	329	0.4836	0.3272	0.7703	0.9093
tox21-pgc-err-p1	1726	0.3383	0.2500	0.7635	0.8967
tox21-elg1-luc-agonist-p1	115	0.4824	0.3729	0.7574	0.8809
tox21-adrb2-antagonist-p1	148	0.5259	0.4153	0.7532	0.9298
tox21-shh-3t3-gli3-agonist-p1	127	0.4317	0.3117	0.7389	0.8934
tox21-rar-viability-p2	283	0.3487	0.2678	0.7305	0.8702
tox21-hse-bla-p1	283	0.4272	0.2561	0.7240	0.8919
tox21-ppard-bla-agonist-p1	128	0.4834	0.3775	0.7060	0.8440
tox21-er-bla-antagonist-p1	544	0.4746	0.2909	0.6958	0.8774
tox21-adrb2-agonist-p1	202	0.5563	0.4219	0.6955	0.8825
tox21-ms-p53-p2	87	0.3323	0.2739	0.6951	0.9067
tox21-gh3-tre-agonist-p1	39	0.6120	0.5315	0.6803	0.9522
tox21-vdr-bla-agonist-p1	128	0.4931	0.4237	0.6637	0.8342
tox21-kiss1r-hek293-p2	256	0.5978	0.4035	0.6341	0.8301
tox21-rt-viability-hepg2-p1-glo_16h_n	93	0.2609	0.1735	0.6032	0.8121
tox21-drd2-agonist-p1	35	0.8114	0.6692	0.5982	0.9047
tox21-nfkb-bla-agonist-p1	67	0.6302	0.4140	0.5302	0.7820
tox21-hdac-p1	319	0.3306	0.2764	0.5301	0.9495
tox21-rt-viability-hepg2-p1-flor_24h_n	137	0.2545	0.1927	0.5251	0.8257
tox21-rt-viability-hepg2-p1-glo_24h_n	96	0.3387	0.2296	0.5231	0.8491
tox21-hre-bla-agonist-p1	172	0.3445	0.2441	0.4973	0.8719
tox21-rt-viability-hepg2-p1-flor_8h_n	81	0.3329	0.2723	0.4626	0.7629
tox21-rt-viability-hepg2-p1-glo_8h_n	83	0.2701	0.1860	0.4279	0.7421
tox21-ms-ache-p2	41	0.5098	0.3476	0.4257	0.8089
tox21-ms-p53-p1	85	0.4509	0.3132	0.4234	0.8542
tox21-rt-viability-hek293-p1-flor_8h_n	51	0.2894	0.2449	0.4190	0.7638
tox21-rt-viability-hepg2-p1-flor_16h_n	111	0.3973	0.3322	0.4159	0.8436
tox21-tshr-antagonist-p1	205	0.3777	0.2679	0.3868	0.7647
tox21-rt-viability-hek293-p1-flor_16h_n	80	0.3863	0.3344	0.3311	0.8140
tox21-trhr-hek293-p1	359	0.4761	0.3349	0.2732	0.7032
tox21-rt-viability-hek293-p1-flor_24h_n	100	0.3703	0.3318	0.1484	0.8643
tox21-kiss1r-wt-p2	54	0.2676	0.2056	-0.1582	0.4470
tox21-fxr-bla-agonist-p2	85	0.5483	0.3928	-0.1857	0.6351
tox21-sbe-bla-agonist-p1	29	0.5550	0.4585	-5.7981	0.3796

Self-supervised pretraining alone yields a strong representation
Without auxiliary supervision, Tox21mer still performed strongly, reaching mechanism macro-F1 0.957 (agonist 0.925, antagonist 0.952, inactive 0.998) and non-mechanism binary-F1 0.966 (active 0.947, inactive 0.986). PR-AUC remained high for all classes, exceeding 0.977 throughout. Relative to the auxiliary-supervised model.

Table S5. Effect of auxiliary supervised losses on branch-probe performance without auxiliary supervision.

	Mech F1				Non-mech F1		
**Configuration**	**Macro Avg**	**Agonist**	**Antagonist**	**Inactive**	**Binary**	**Active**	**inactive**
Tox21mer (with auxiliary supervision)	0.9848	0.9779	0.9787	0.9978	0.9856	0.9778	0.9856
Tox21mer (no auxiliary supervision)	0.9568	0.9246	0.9522	0.9937	0.9664	0.9472	0.9855
Difference (supervision − none)	+0.028	+0.053	+0.027	+0.004	+0.019	+0.031	+0.001

Additional Ablation Intervention
In the main text, we reported three blocks of ablations designed to decouple the contributions of response values, response ordering, and assay context to the backbone's CLS representation (**Table S6**). To assess the contribution of assay-context metadata to probe performance, we repeated the Phase 2a evaluation with the six categorical context features (protocol name, target category, cell type, assay mode, technology, and ratio flag) zeroed at every dose–response token during the frozen-backbone forward pass, so that the CLS embedding was derived solely from the response values and log-dose inputs. This ablation produced mechanism macro-F1 of 0.986 and non-mechanism binary-F1 of 0.994, essentially indistinguishable from the full-context baseline (0.985 and 0.986, respectively) (**Table S6**). Per-class PR-AUC remained at or above 0.997 on all four classes, and the confusion matrices showed no systematic shift in error pattern. The near-zero effect of removing assay context is consistent with the observation that, within each protocol, the response-value distribution alone is already highly discriminative for outcome classification, protocol identity is largely redundant once the model observes the response magnitudes. This result does not imply that assay context is uninformative in general; it indicates that, for the branch-probe task as posed, the response values carry sufficient signal. Context may become more important for cross-protocol transfer or for tasks where protocol identity is not implicitly recoverable from the response distribution.

Table S6. Single-factor ablation with zero-context inputs applied during Phase 1 pretraining.

Ablation flag	Mech F1	Non-mech F1	Active recall	Agonist recall	Antagonist recall
None (baseline)	0.9848	0.9935	0.9856	0.9750	0.9815
`--zero-dr-assay-ctx`	0.9857	0.9861	0.9877	0.9816	0.9850

Table S7. Transformer vs MLP baseline performance on the test set, using identical branch-probe supervision, label masking, and metrics. The 2-layer MLP on raw `resp_norm` plus `assay_ctx` matches the transformer to within one F1 point on both heads.

Model	Features	Mech F1	Non-mech F1	Score
Tox21mer	Backbone CLS	0.985	0.986	0.991
MLP (stats only)	8 summary stats	0.846	0.965	0.906
MLP (stats + ctx)	8 stats + `assay_ctx`	0.948	0.989	0.969
MLP (raw only)	15 Raw `resp_norm`	0.910	0.981	0.945
MLP (raw + ctx)	Raw `resp_norm` + `assay_ctx`	0.983	0.996	0.990

Additional Discussion on Ablations

The contrast between Phases 2b and 2d indicates a marked asymmetry: evaluating a clean-trained probe on shuffled inputs reduced performance much more than evaluating a shuffled-trained probe on clean inputs. This pattern suggests that the observed drop under shuffling is driven mainly by probe-level distribution shift rather than by complete loss of predictive information in the backbone representation. Consistent with this interpretation, probes trained on shuffled curves recovered near-baseline performance, indicating that substantial outcome-relevant information is retained even when dose–response ordering is disrupted.

The MLP comparison supports a similar conclusion. Raw-response MLPs, especially when combined with assay context, achieved probe performance close to that of the transformer. Together, these results suggest that the probe tasks rely heavily on response-value distributions and assay context, while being less sensitive to fine-grained within-curve ordering. This does not diminish the broader utility of Tox21mer, but it does clarify the specific aspects of representation quality captured by the probe-based evaluation.

Additional Discussion on Supervision

We verified that probe-evaluation performance does not arise from circular reuse of pretraining supervision, we additionally pretrained Tox21mer with all auxiliary supervised loss weights set to zero (masked-response reconstruction only). The resulting purely self-supervised backbone retained mechanism macro-F1 of 0.957 and non-mechanism binary-F1 of 0.986, with per-class PR-AUC at or above 0.977 on every class. Auxiliary supervision contributes 1–6 F1 points of improvement, concentrated on the minority active classes, consistent with the interpretation that auxiliary terms sharpen minority-class decision boundaries beyond what reconstruction alone recovers. Probe metrics therefore measure compound-level generalization rather than retention of supervised pretraining signal. We therefore interpret the probe metrics as measuring generalization across held-out compounds, not simple memorization of the auxiliary task family. Second, pretraining objectives that explicitly reward shape-aware representations, for example, contrastive objectives that distinguish permutations of the same response bag,

or auxiliary regression of fitted Hill-slope parameters, may produce embeddings that are more informative on tasks where shape is dispositive, such as mechanism-of-action classification across novel receptor classes. Third, adapting Tox21mer to related high-throughput assay resources, including ToxCast and recent concentration-response extensions of the Tox21 10K panel, would allow the representation to be exercised across a broader chemical and assay space and would clarify which aspects of the current model are dataset-specific and which generalize.
